# Durability of the moderate-to-heavy-intensity transition is related to the effects of prolonged exercise on severe-intensity performance

**DOI:** 10.1007/s00421-024-05459-6

**Published:** 2024-03-28

**Authors:** Kate Hamilton, Andrew E. Kilding, Daniel J. Plews, Mathew J. Mildenhall, Mark Waldron, Thanchanok Charoensap, Tobias H. Cox, Matthew J. Brick, Warren B. Leigh, Ed Maunder

**Affiliations:** 1 Sports Performance Research Institute New Zealand, Auckland University of Technology, Auckland, New Zealand; 2 High Performance Sport New Zealand, Auckland, New Zealand; 3 A-STEM Centre, College of Engineering, Swansea University, Swansea, UK; 4 School of Science and Technology, University of New England, Armidale, NSW Australia; 5 Orthosports North Harbour, AUT Millennium, Auckland, New Zealand

**Keywords:** Durability, Exercise, Muscle

## Abstract

**Purpose:**

Power output at the moderate-to-heavy-intensity transition decreases during prolonged exercise, and resilience to this has been termed ‘durability’. The purpose of this study was to assess the relationship between durability and the effect of prolonged exercise on severe-intensity performance, and explore intramuscular correlates of durability.

**Methods:**

On separate days, 13 well-trained cyclists and triathletes (V̇O_2_peak, 57.3 ± 4.8 mL kg^−1^ min^−1^; training volume, 12 ± 2.1 h week^−1^) undertook an incremental test and 5-min time trial (TT) to determine power output at the first ventilatory threshold (VT_1_) and severe-intensity performance, with and without 150-min of prior moderate-intensity cycling. A single resting *vastus lateralis* microbiopsy was obtained.

**Results:**

Prolonged exercise reduced power output at VT_1_ (211 ± 40 vs. 198 ± 39 W, ∆ -13 ± 16 W, ∆ -6 ± 7%, *P* = 0.013) and 5-min TT performance (333 ± 75 vs. 302 ± 63 W, ∆ -31 ± 41 W, ∆ -9 ± 10%, *P* = 0.017). The reduction in 5-min TT performance was significantly associated with durability of VT_1_ (r_s_ = 0.719, *P* = 0.007). Durability of VT_1_ was not related to *vastus lateralis* carnosine content, citrate synthase activity, or complex I activity (*P* > 0.05).

**Conclusion:**

These data provide the first direct support that durability of the moderate-to-heavy-intensity transition is an important performance parameter, as more durable athletes exhibited smaller reductions in 5-min TT performance following prolonged exercise. We did not find relationships between durability and *vastus lateralis* carnosine content, citrate synthase activity, or complex I activity.

## Introduction

Performance outcomes in stochastic endurance events, such as road cycling, are often determined by the ability to produce high work outputs in the severe-intensity domain following multiple hours of exercise, primarily performed in the moderate-intensity domain (Fernández-García et al. 2000; Sanders et al. 2019). Physiological profiling characteristics are estimated in well-rested athletes during routine laboratory assessments and used for the assessment of performance capabilities, within-session intensity regulation, and monitoring training load and adaptation (Maunder et al. 2021b). Prolonged exercise elicits progressive physiological changes, such as increased core and muscle temperature (Febbraio et al. 1994), depletion of endogenous fuel stores (Gonzalez et al. 2016; Areta and Hopkins 2018; Stokie et al. 2023), accumulation of muscle damage (Stevens et al. 2018), and cellular stress (Morton et al. 2009; Peake et al. 2017). Consequently, physiological profiling characteristics, such as work output at the moderate-to-heavy (Stevenson et al. 2022b) and heavy-to-severe (Clark et al. 2018, 2019a, 2019b)-intensity transitions, gross cycling efficiency (Passfield and Doust 2000; Hopker et al. 2017) and the peak rate of oxygen consumption (V̇O_2_peak) (Brownstein et al. 2022) may, in some instances, degrade during prolonged exercise. ‘Durability’ is defined as an individual’s resilience to deteriorations in physiological profiling characteristics during prolonged exercise, and has been proposed as a key endurance performance parameter (Maunder et al. 2021b). However, the influence of durability on performance outcomes has not been well-characterised.

Like physiological profiling characteristics, severe-intensity performance decreases with prolonged exercise (Spragg et al. 2023a, b). Durability of the intensity domain transitions may promote resilience to the effects of prolonged exercise on severe-intensity performance. As power output at the moderate-to-heavy-intensity transition decreases during prolonged exercise (Stevenson et al. 2022b), an initially moderate-intensity power output may drift into the heavy-intensity domain. Heavy-intensity exercise elicits distinct physiological responses compared to the moderate-intensity domain, such as greater plasma K^+^ accumulation, reduced intramuscular pH, and phosphocreatine depletion (Black et al. 2017). Therefore, greater time spent in the heavy-intensity domain may result in reduced subsequent capacity for work outputs in the severe-intensity domain. This ‘domain drift’ may also be present during prolonged exercise initially in the heavy intensity domain, where exercise may drift into the severe domain. Accordingly, better durability of the intensity transitions may promote resilience to the effects of prolonged exercise on subsequent severe-intensity performance. However, the relationship between these variables has not been assessed.

The effect of prolonged exercise on subsequent severe-intensity performance may also be related to glycogen depletion. Glycogen depletion may impair Na^+^,K^+^-ATPase pump activity, and therefore the ability to regulate K^+^ homeostasis and maintain muscle contractile function (Jensen et al. 2020). Specifically, Na^+^,K^+^-ATPase function appears dependent on intramyofibrillar glycogen (Nielsen et al. 2022), and intramyofibrillar glycogen depletion has been implicated in impaired muscle contractile function (Nielsen et al. 2009, 2014; Ørtenblad et al. 2011, 2013). Furthermore, recent work in mouse muscle suggests glycogen depletion increases the number of inexcitable muscle fibres unable to contribute to force and power production, and therefore performance (Cairns and Renaud 2023). Therefore, it is plausible that athletes capable of oxidising fat at high rates to preserve glycogen during submaximal exercise may be better able to maintain severe-intensity performance following prolonged exercise. Indeed, fat oxidation rates during submaximal exercise have been associated with durability of the heavy-to-severe-intensity transition and severe-intensity performance (Spragg et al. 2023b). The capacity for fat oxidation during exercise has been quantified using the peak fat oxidation rate (PFO) observed during an incremental exercise test (Maunder et al. 2018, 2023), and PFO has been related to endurance performance outcomes (Frandsen et al. 2017; Maunder et al. 2022). However, the relationship between PFO and the effect of prolonged exercise on severe-intensity performance has not been assessed.

Similarly, the physiological determinants of durability are not well-explored. Plausibly, skeletal muscle fibre type composition may influence durability, as type I fibres are more fatigue resistant (Thorstensson and Karlsson 1976). Likewise, possessing a larger mitochondrial pool may spread the oxidative burden of a given work rate, and therefore reduce negative effects on the function of individual mitochondria during prolonged exercise (Sahlin et al. 2010; Trewin et al. 2017; Layec et al. 2018; Lewis et al. 2021). This may promote durability by delaying deteriorations in mitochondrial function, and therefore the oxidative capacity of muscle. Heat shock protein 70 (HSP70) availability may also relate to durability. HSP70 is an intracellular chaperone involved in managing protein aggregation and cellular stress (Krüger et al. 2019). Greater HSP70 abundance may, therefore, augment the capacity to manage the cellular stress generated during prolonged exercise, and therefore promote durability. Understanding the mechanistic determinants of durability may allow for the development of targeted interventions to improve it.

Therefore, the primary aims of the present investigation were to: (i) determine if durability of the moderate-to-heavy transition is related to the magnitude of prolonged exercise-induced reductions in severe-intensity performance, (ii) assess the relationship between prolonged exercise-induced reductions in severe-intensity performance and PFO and (iii) quantify relationships between various intramuscular characteristics and durability of the moderate-to-heavy-intensity transition. We hypothesised that cyclists who have greater durability of the moderate-to-heavy-intensity transition would be more resilient to the effect of prolonged exercise on severe-intensity performance, that resilience to the effects of prolonged exercise on severe-intensity performance would be related to PFO, and that durability of the moderate-to-heavy-intensity transition would be related to various oxidative properties of skeletal muscle.

## Methods

### Ethical approval

The study was performed in accordance with the Declaration of Helsinki, 2013. The Auckland University of Technology Ethics Committee approved all procedures (22/163), and all participants provided written informed consent prior to participation. This study was not registered in a database. Raw data are available upon request.

### Participants

Thirteen well-trained endurance cyclists and triathletes completed the present investigation (eleven males, two females; age, 29 ± 7; height, 182.6 ± 8 cm; mass, 78.1 ± 11.7 kg; V̇O_2_peak, 57.3 ± 4.8 mL kg^−1^ min^−1^; training volume, 12 ± 2.1 h week^−1^). A-priori calculations indicated that 15 participants were required to detect a significant bivariate correlation of *r* = 0.6, assuming a null hypothesis correlation of *r* = 0, and a one-tailed test, with 80% statistical power and a type I error rate of 0.05. All participants were free of recent (< 3 months) illness and musculoskeletal injury, free of cardiovascular disease, and training > 8 h week^−1^ in endurance cycling, with a peak oxygen uptake > 55 mL kg^−^1 min^−1^ and self-reported best-effort 20-min power output of > 3.5 W kg^−1^. All participants provided written informed consent. Three participants dropped out after the first visit due to the inclusion criteria of remaining free of illness for > 3 months.

### Study design

**Fig 1:**
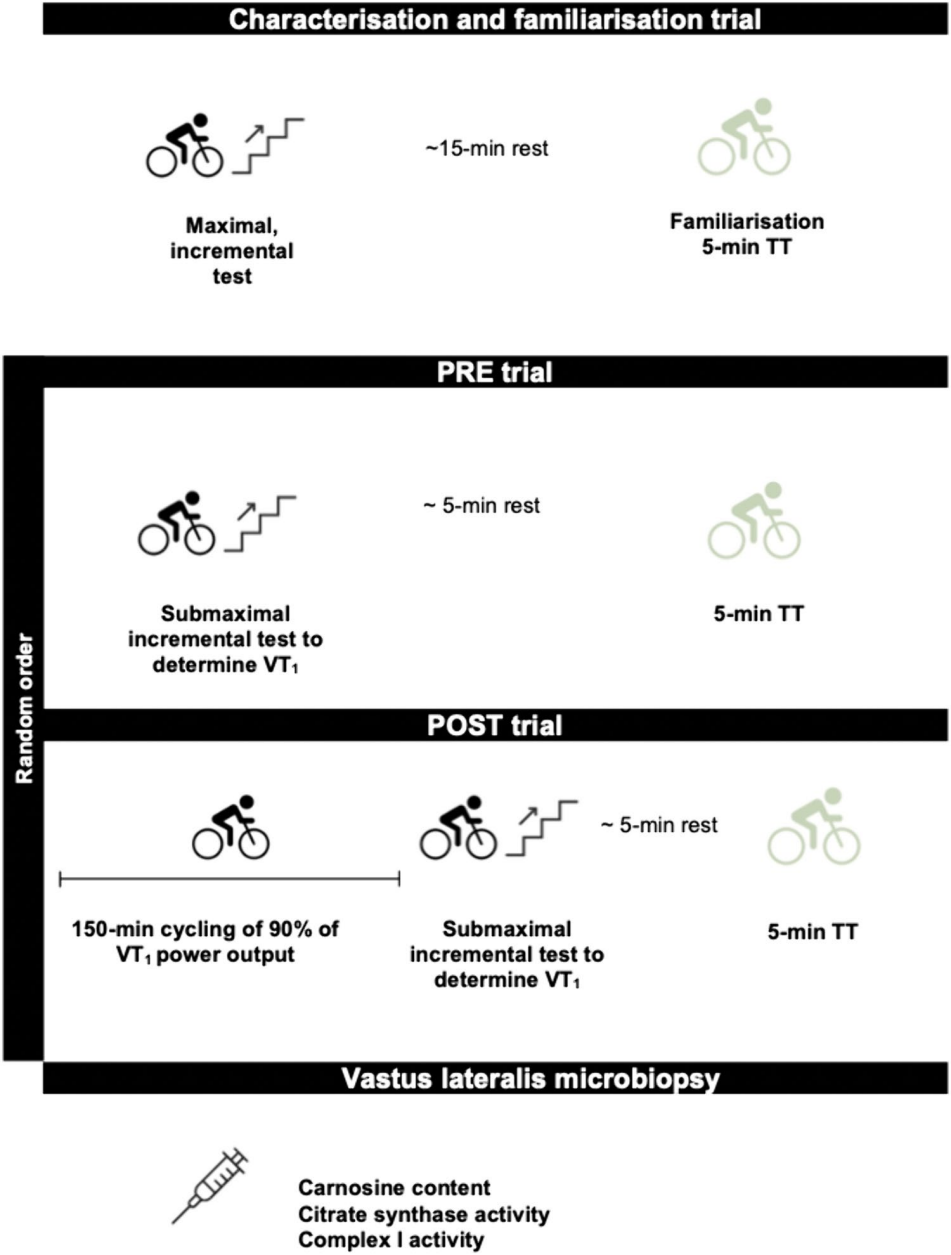
Schematic of the study design. TT, time trial; VT1, first ventilatory threshold

Participants visited the laboratory on four separate occasions, ~ 5–14 days apart, at ~ 6:00 am (Fig. 1). The first visit was a characterisation trial, involving an incremental cycling test for estimation of power output at the first ventilatory threshold (VT_1_) for use in subsequent trials and peak oxygen uptake (V̇O_2_peak), and a familiarisation 5-min time trial. The second and third visits took place in random, counterbalanced order and involved either (i) PRE: a submaximal incremental test to determine the moderate-to-heavy-intensity transition, and performance and V̇O_2_peak in a 5-min time trial, or (ii) POST: 150 min of cycling at 90% of the first ventilatory threshold (VT_1_) power output estimated in the characterisation trial, followed by the submaximal incremental test to determine the moderate-to-heavy-intensity transition, and performance and V̇O_2_peak in a 5-min time trial. The 5-min time trials were used to mimic a decisive effort in a road cycling event, and because these fall within the severe-intensity domain (Jones et al. 2019). Fixed-duration cycling time trials also produce reliable data, particularly in trained cyclists following a familiarisation trial (Hopkins et al. 2001; Paton and Hopkins 2001; Leo et al. 2022). The remaining visit was for a resting *vastus lateralis* microbiopsy.

### Characterisation trial

Participants reported to the laboratory for an initial incremental cycling test and 5-min familiarisation time trial, after having fasted overnight for ~ 10 h and having ingested 1–2 L of plain water before arrival. After providing written informed consent, height and mass were determined. Cycling commenced with a 5-min warm-up at 100 W on personal road bicycles mounted to a direct-drive smart indoor trainer (Kickr, Wahoo Fitness, GA, USA). Subsequently, the incremental cycling test began at 95 W, with the power output increasing by 35 W every 3 min. Expired gases and heart rate were collected continuously using indirect calorimetry (TrueOne 2400, ParvoMedics, UT, USA) and a chest-strap heart rate monitor (Polar Electro Oy, Kempele, Finland). When the respiratory exchange ratio reached 1.0, power output was increased by 35 W every minute until volitional exhaustion. The V̇O_2_peak was accepted as the highest 15-s average V̇O_2_, and VT_1_ was identified as the first V̇O_2_ breakpoint in the V̇O_2_ vs. V̇_E_^.^V̇O_2_^−1^ relationship. This V̇O_2_ was converted to power output by linear regression of the V̇O_2_ vs. power output relationship, using the last minute of V̇O_2_ data from each 3-min stage. The last minute of expired gas data in each 3-min stage was also used to quantify whole-body rates of carbohydrate and fat oxidation using standard equations (Eq. 1) (Jeukendrup and Wallis 2005). The highest observed rate of whole-body fat oxidation was identified as the peak fat oxidation rate (PFO) (Maunder et al. 2022).

1 $$\begin{gathered} {\text{Carbohydrate oxidation rate }}\left( {{\text{g min}}^{{ - {1}}} } \right)\, = \,{4}.{21}0\, \times \,{\dot{\text{V}}\text{CO}}_{{2}} {-}{2}.{962}\, \times \,{\dot{\text{V}}\text{O}}_{{2}} \hfill \\ {\text{Fat oxidation rate }}\left( {{\text{g min}}^{{ - {1}}} } \right)\, = \,{1}.{695}\, \times \,{\dot{\text{V}}\text{O}}_{{2}} {-}{1}.{7}0{1}\, \times \,{\dot{\text{V}}\text{CO}}_{{2}} \hfill \\ \end{gathered}$$

where V̇O2 and V̇O2 are in L.min^-1^

Following completion of the incremental test, participants rested for 15 min before completing a 5-min performance time trial at maximal effort, with the goal of achieving the highest possible average power output. Expired gases and heart rate were collected throughout; however, participants were blinded to all data other than elapsed time. Verbal encouragement was not provided to control the influence of this variable on performance outcomes (McCormick et al. 2015). We have refrained from providing verbal encouragement during time trials previously (Maunder et al. 2021a). The importance of providing a maximum effort during time trials was stressed to participants at the beginning of the study, and participants were reminded of this ahead of each time trial. The highest 15-s average of V̇O_2_ was accepted as the time trial V̇O_2_peak. If this value exceeded the V̇O_2_peak achieved during the maximal, incremental cycling test, it was used as the characterisation trial V̇O_2_peak. Following the time trial, participants were provided with blank 7-d exercise and 2-d diet record sheets, which were completed in advance of the second trial, and replicated in advance of the third trial.

### Visits two and three: PRE and POST assessments

Participants returned to the laboratory 5–14 days following the characterisation trial to complete the first of the two subsequent trials. Participants arrived having consumed a standardised breakfast containing ~ 2 g kg^−1^ of carbohydrate and ~ 800 mL of water 1 h beforehand. Participants were fitted with a chest-strap heart rate monitor, and a wireless near-infrared spectroscopy device for estimation of muscle oxygenation (S_m_O_2_) on their right leg (Moxy Monitor, Fortiori Design LLC, Hutchinson, MN, USA). The device was placed over the mid-belly of the *vastus lateralis*, half the distance between the tibial tuberosity and greater trochanter. The precise site of the device was recorded such that it could be repeated in the remaining trial. Heart rate and muscle oxygenation were measured continuously throughout the trial.

The PRE and POST trials began with a 5-min warm-up at 100 W. Following warm-up, participants cycled for 150 min at 90% of their estimated VT_1_ power output in the POST but not PRE trial, with expired gases collected for 4 min every 15 min. Expired gases were used to quantify rates of whole-body rates of energy expenditure, carbohydrate oxidation, and fat oxidation during the 150-min preload using standard equations (Jeukendrup and Wallis 2005). In POST, participants consumed 150 mL of water every 15 min in a solution made with electrolyte mix (LMNT) containing 125 mg Na^+^, 25 mg K^+^, and 7.5 mg Mg^2+^ during the first 120 min of the 150-min preload.

Subsequently, the moderate-to-heavy-intensity transition was estimated precisely using a five-step incremental test with continuous collection of expired gases. The first step began 50 W below the VT_1_ power output estimated in the first laboratory visit, and increased by 25 W every 4 min, such that the fifth and final step was 50 W above the VT_1_ power output estimated in the first laboratory visit. The moderate-to-heavy-intensity transition power output was estimated using the methods described for determining VT_1_ in the first laboratory visit, but with greater precision given the greater density of data around the transition. This method has been used to estimate the moderate-to-heavy-intensity transition previously, producing similar results to blood lactate-derived measurements (Stevenson et al. 2022b). Following the five-step incremental test, participants cycled at 100 W for 5 min before completing a 5-min performance time trial according to the procedures described above. The effect of prolonged exercise on the moderate-to-heavy-intensity transition power output, 5-min time trial performance, and V̇O_2_peak during the 5-min time trial was determined by subtracting PRE from POST values. We used this exercise protocol as we previously observed reduced power output at the moderate-to-heavy-intensity transition after 150 min of initially moderate-intensity cycling (Stevenson et al. 2022b), and to simulate a road cycling event, in which a severe-intensity effort near the finish may follow a longer period of lower intensity cycling (Fernández-García et al. 2000; Sanders et al. 2019).

### Visit four: resting vastus lateralis microbiopsy

Approximately 5–14 days following the third visit, participants returned to the laboratory having consumed breakfast and having refrained from vigorous exercise for 24 h. A ~ 15–30 mg resting microbiopsy sample was obtained from the mid-belly of the *vastus lateralis* of the dominant leg, ~ 10–15 cm above the patella. Local anaesthesia was applied to the skin and superficial muscle fascia. A microbiopsy needle was then inserted into the mid-belly of the *vastus lateralis* to a depth of ~ 2 cm to recover ~ 20–40 mg of tissue using a spring-loaded mechanism (14G Ultimate Biopsy Needle, Zamar Care, Croatia). Muscle tissue was immediately frozen on dry ice and stored at −80 °C until further analysis.

### Muscle analyses

Frozen muscle was cut and rinsed using cold phosphate-buffered saline (PBS) and then suspended to ~ 25 mg^.^mL^−1^ in PBS. Samples were then ground manually and thoroughly using a pre-cooled Dounce homogeniser. Homogenate was solubilised in extraction buffer (ab260490, Abcam®) to ~ 5 mg mL^−1^ and incubated on ice for 20 min prior to centrifugation at 16,000 g for 10 min at 4 °C. Supernatant was extracted and stored at −80 °C prior to further analysis. Supernatant was thawed and assayed in duplicate for carnosine concentration (MBS721162, MyBioSource^®^; our laboratory-specific coefficient of variation [CV], 12.6%), citrate synthase enzyme activity (ab119692, Abcam^®^; CV, 11.4%), and complex I enzyme activity (ab109721, Abcam^®^; CV, 12.1%). All outcome measures were expressed relative to sample protein concentration using a Bradford assay, performed in triplicate (ab102535, Abcam^®^; CV, 6.1%). Carnosine concentrations, as assessed by ^1^H-magnetic resonance spectroscopy, have been related to fibre type profile (Baguet et al. 2011). Lower carnosine concentrations are observed in type I compared to type II skeletal muscle fibres (Harris et al. 1998). We, therefore, used our measures of muscle carnosine as an indicator of muscle fibre type profile. CS and complex I activities are related to mitochondrial protein content (Larsen et al. 2012). We, therefore, used our measures of CS and complex I activities as markers of mitochondrial protein content.

### Statistical analyses

Data are expressed as mean ± standard deviation, unless otherwise stated. Normality of datasets was assessed using Shapiro–Wilk tests. Simple PRE vs. POST comparisons of the moderate-to-heavy-intensity transition power output, V̇O_2_peak, and time trial performance were made using paired *t* tests or Wilcoxon signed-rank tests, depending on normality, and used to verify the effect of prolonged exercise on these parameters. The effect of time on whole-body rates of energy expenditure, carbohydrate oxidation, fat oxidation, S_m_O_2_, and heart rate during the 150 min preload in POST was analysed using one-way repeated measures analyses of variance.

Bivariate relationships between (i) the magnitude of the PRE vs. POST change in moderate-to-heavy-intensity transition power output and the magnitude of the PRE vs. POST change in time trial performance, (ii) the magnitude of the PRE vs. POST change in V̇O_2_peak and the magnitude of the PRE vs. POST change in time trial performance, (iii) the magnitude of the PRE vs. POST change in time trial performance and PFO, (iv) the magnitude of the PRE vs. POST change in moderate-to-heavy-intensity transition power output and skeletal muscle characteristics were assessed using Pearson’s or Spearman’s rank-order correlation coefficients (depending on normality), and expressed with 95% confidence intervals. The strength of correlations were assessed according to the following qualitative criteria: < 0.10, trivial; 0.10–0.29, small; 0.30–0.49, moderate; > 0.50, large (Cohen 1992). The PRE and POST values for S_m_O_2_ at the moderate-to-heavy-intensity transition were compared using paired *t* tests or Wilcoxon signed-rank tests, depending on normality, and intra-class correlation coefficients and coefficient of variation statistics were computed. All analyses were performed in GraphPad Prism Version 9.3.1. Significance was inferred when *P* ≤ 0.05.

## Results

### Prolonged exercise phase

**Fig 2:**
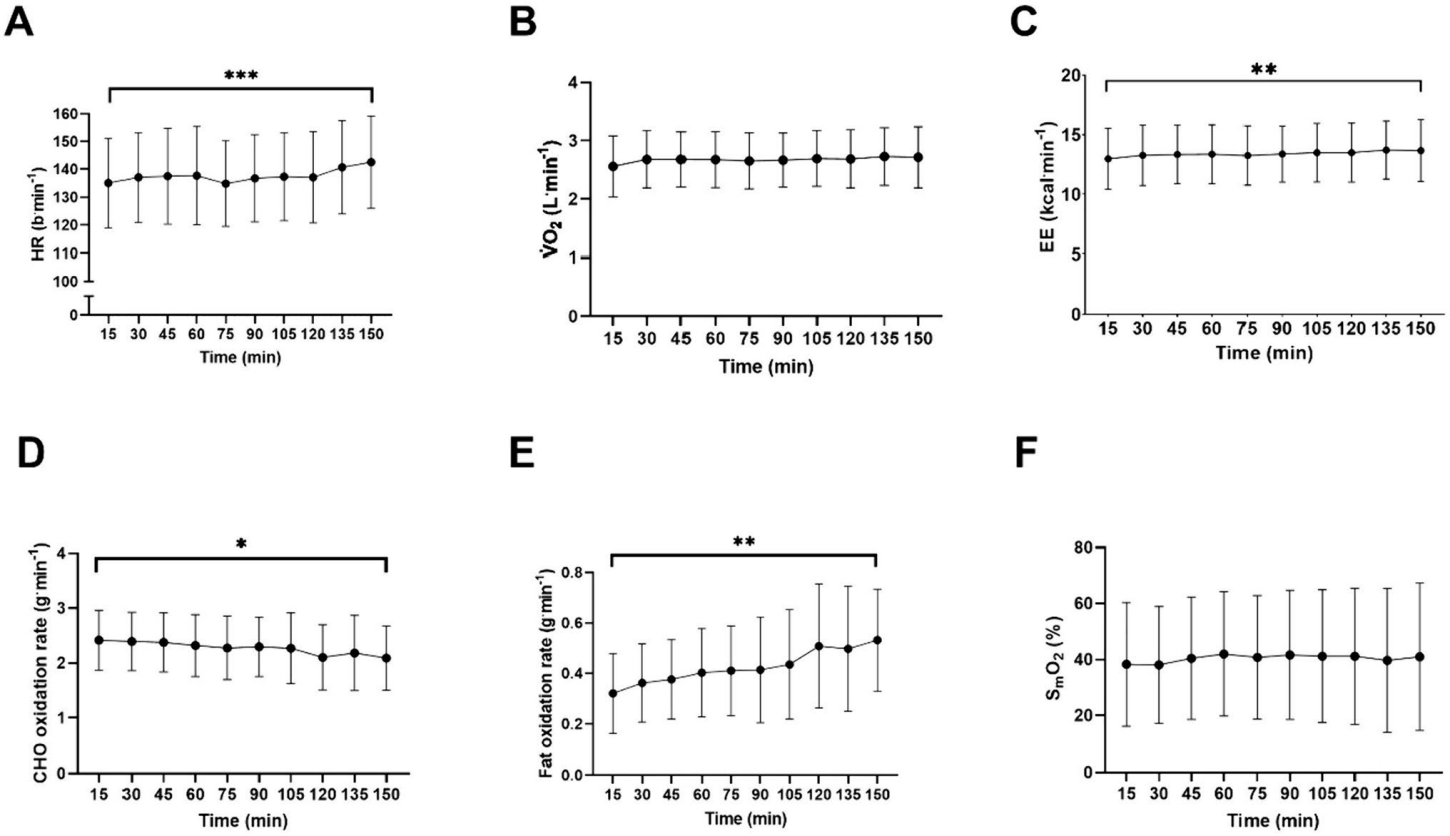
A Heart rate (HR), B rate of oxygen consumption (V̇O2), C energy expenditure (EE), D carbohydrate (CHO) oxidation rate, E fat oxidation rate and F muscle oxygen saturation (SmO2) during the prolonged phase of the POST trial. * denotes *P* ≤ 0.05, ** denotes *P* ≤ 0.01, *** denotes *P* ≤ 0.001

The estimated power output at VT_1_ in the initial assessment was 208 ± 30 W. The 150-min prolonged phase in POST was, therefore, completed at 187 ± 27 W. During the prolonged phase, there was an effect of time on heart rate, EE, carbohydrate oxidation, and fat oxidation (*P* < 0.05). Significant effects of time on V̇O_2_ and S_m_O_2_ were not observed (Fig. 2).

### Effects of prolonged exercise

**Fig 3:**
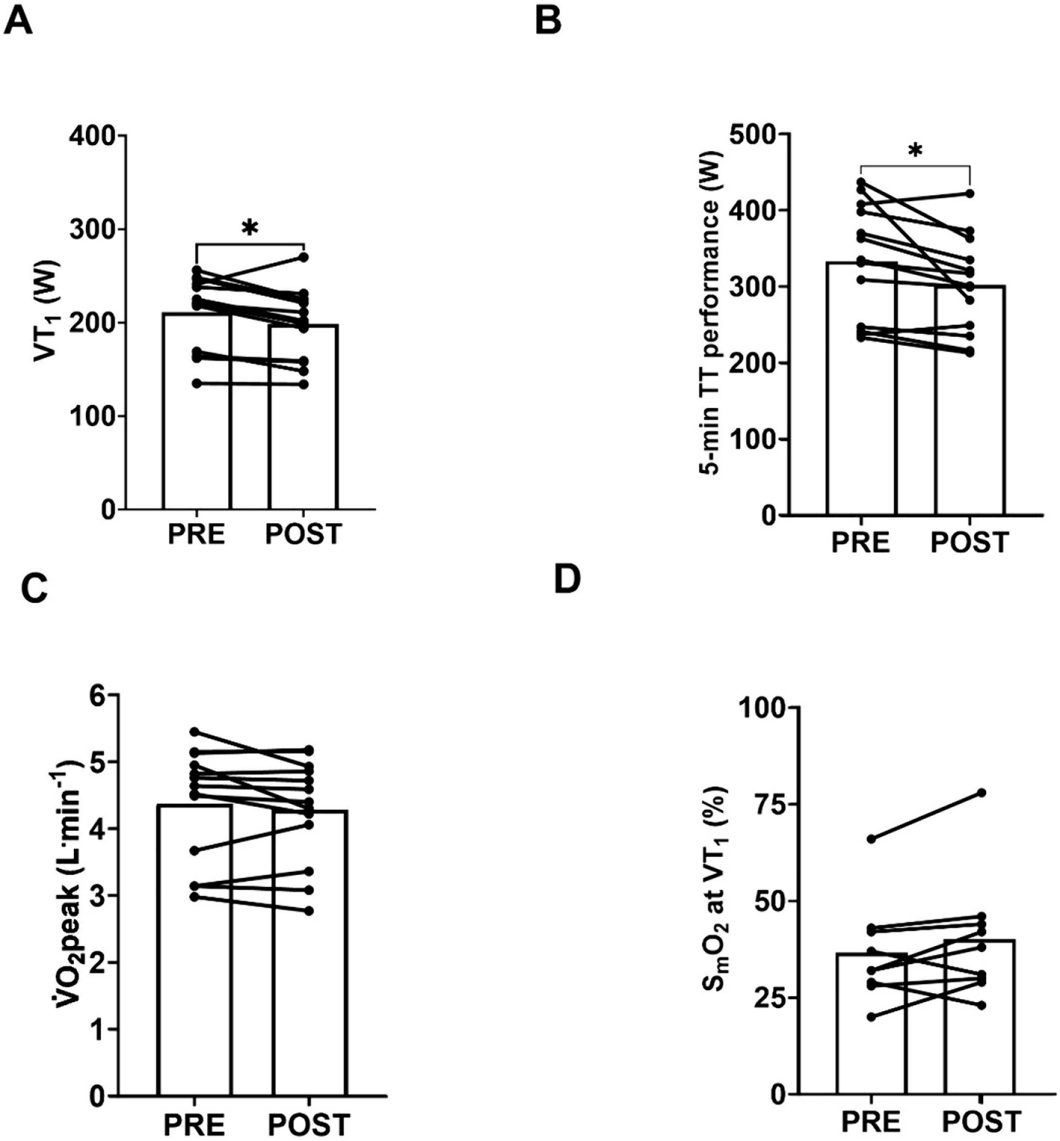
A Power output at the first ventilatory threshold (VT1), B 5-min time trial (TT) performance, C peak rate of oxygen consumption (V̇O2peak) and D muscle oxygen saturation (SmO2) at VT1 in the PRE and POST assessments. * denotes *P* ≤ 0.05

Power output at VT_1_ (211 ± 40 W vs. 198 ± 39 W, ∆ -13 ± 16 W, ∆ -6 ± 7%, *P* = 0.013) and 5-min time trial performance (333 ± 75 W vs. 302 ± 63 W, ∆ -31 ± 41 W, ∆ -9 ± 10%, *P* = 0.017) significantly decreased from PRE to POST. The V̇O_2_peak (4.37 ± 0.85 vs. 4.28 ± 0.78 L^.^min^−1^, *P* = 0.252) and S_m_O_2_ at VT_1_ (37 ± 13 vs. 40 ± 16%, *P* = 0.139) were not significantly different between PRE and POST (Fig. 3). The within-subject CV for S_m_O_2_ at VT_1_ in PRE and POST was 13.0%, with an intra-class correlation of 0.874.

### Correlational analyses

**Fig 4:**
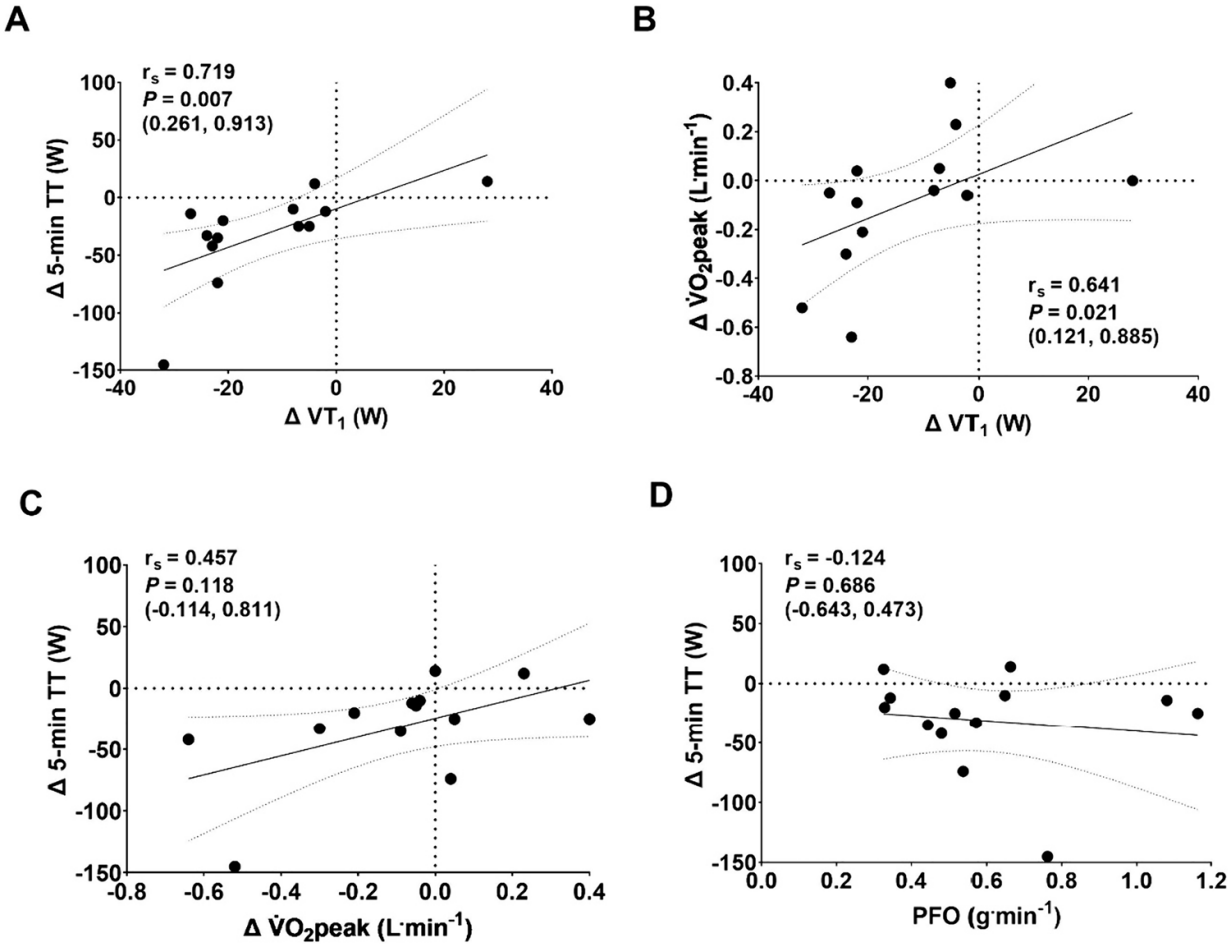
Relationships between A PRE-to-POST changes in VT1 and 5-min time trial (TT) performance, B PRE-to-POST changes VT1 and V̇O2peak, C PRE-to-POST changes in V̇O2peak and 5-min TT performance, and D peak fat oxidation rate (PFO) and PRE-to-POST changes in 5-min TT performance. Data are presented as Spearman’s rank-order correlation coefficients (rs) with *P* values and 95% confidence intervals.

**Fig 5:**
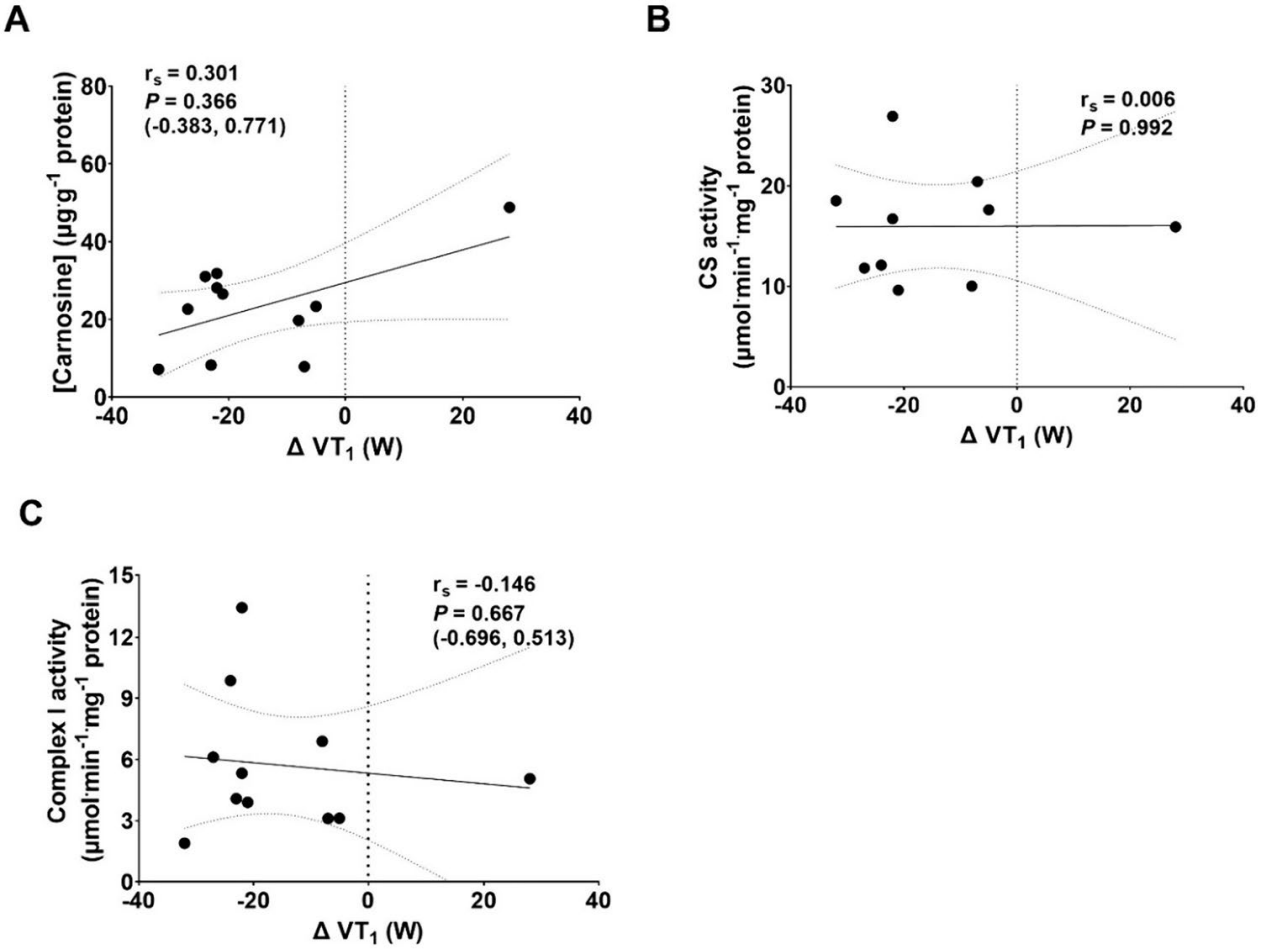
Relationships between the PRE-to-POST change in VT1 power output and vastus lateralis: A carnosine concentration, B citrate synthase (CS) activity and C complex I activity. Data are presented as Spearman’s rank-order correlation coefficients (rs) with *P* values and 95% confidence intervals

The change in power output at VT_1_ from PRE to POST was strongly associated with PRE-to-POST change in 5-min time trial power output and V̇O_2_peak (*P* < 0.05). The PRE-to-POST change in V̇O_2_peak was not significantly associated with the change in 5-min time trial power output, nor was PFO (Fig. 4). No significant relationships were observed between the PRE-to-POST change in power output at VT_1_ and *vastus lateralis* carnosine concentration (24.0 ± 12.2 µg^.^g^−1^ protein), CS activity (16.1 ± 5.1 µmol ^−1^ min^−1^ mg^−1^ protein), or complex I activity (5.8 ± 3.2 µmol ^−1^ min^−1^ mg^−1^ protein) (Fig. 5).

## Discussion

Our primary observations were that: (i) prolonged exercise-induced reductions in severe-intensity time trial performance were strongly related to durability of the moderate-to-heavy-intensity transition, (ii) prolonged exercise-induced reductions in severe-intensity time trial performance were not related to PFO, (iii) no relationships were observed between *vastus lateralis* CS activity, complex I activity, or carnosine concentration and durability of the moderate-to-heavy-intensity transition. These observations provide the first direct support for the hypothesis that durability of the moderate-to-heavy-intensity transition is an important endurance performance parameter, and therefore further support that durability of the moderate-to-heavy-intensity transition might be monitored at an individual level. The physiological determinants of durability remain to be identified.

In line with our hypothesis and previous work (Stevenson et al. 2022a; Spragg et al. 2023a, b), prolonged exercise led to a reduction in power output at the moderate-to-heavy-intensity transition and 5-min time trial performance (Fig. 3). Our novel observation is that those exhibiting larger reductions in power output at the moderate-to-heavy-intensity transition with prolonged exercise exhibited the largest reductions in 5-min time trial performance (Fig. 3). The strong relationship between the effects of prolonged exercise on the moderate-to-heavy-intensity transition and 5-min time trial performance could plausibly be mechanistically related. Athletes demonstrating greater reductions in power output at the moderate-to-heavy-intensity transition likely spent more time in the heavy domain during the prolonged phase. Heavy-intensity exercise results in greater extracellular K^+^ accumulation than moderate-intensity exercise (Black et al. 2017). Extracellular K^+^ accumulation depresses muscle force production, and therefore induces fatigue in vitro (Cairns et al. 1997; de Paoli et al. 2007). It is, therefore, plausible the more durable athletes were better able to maintain 5-min time trial performance due to better ability to maintain K^+^ homeostasis. However, given an incremental exercise test and 5-min recovery period occurred between the end of the prolonged phase and the 5-min time trial, it is possible that extracellular K^+^ concentrations were restored (Mohr et al. 2011). We suggest that this mechanism is interrogated in studies with measurement of plasma and interstitial K^+^ concentrations.

Second, prolonged exercise-induced reductions in 5-min time trial performance were not related to PFO (Fig. 4d). As muscle glycogen is an important fuel for high-intensity exercise (Vigh-Larsen et al. 2022), and glycogen availability is implicated in muscle fibre excitability and sensitivity to K^+^ disturbance (Cairns and Renaud 2023), we hypothesised that athletes with a higher PFO would better maintain muscle glycogen availability during the prolonged phase, and that this would be favourable for mitigating the fatiguing effects of K^+^ disturbances and maintaining muscle fibre excitability during the subsequent 5-min time trial. Despite the lack of association between the effect of prolonged exercise on 5-min time trial performance and PFO, it remains possible that glycogen availability may at least partially mediate the effects of prolonged exercise on severe-intensity performance. Although PFO during incremental exercise relates to fat oxidation rates during prolonged exercise (Maunder et al. 2022), PFO is not a direct measure of glycogen utilisation or availability. In support, fat oxidation rates during submaximal exercise have been associated with durability of the heavy-to-severe-intensity transition and severe-intensity performance (Spragg et al. 2023b). We, therefore, recommend that future studies interrogate the relationship between the effects of prolonged exercise on severe-intensity performance and muscle glycogen availability using direct measures of glycogen content, ideally with subcellular analyses.

In contrast to our hypothesis, durability of the moderate-to-heavy-intensity transition was not related to *vastus lateralis* carnosine concentration, citrate synthase activity, or complex I activity (Fig. 5). The absence of relationships between these variables and durability of the moderate-to-heavy-intensity transition may indicate that these variables are not mechanistically related, or may be due to the variability in these outcome measures, relatively low sample size, and/or relatively homogenous participant group. We did attempt to measure HSP70 in our muscle samples, but unfortunately this assay did not produce usable results (measured concentrations exceeded the detectable range of the assay). Due to budgetary constraints, we were unable to repeat the assay. We recommend that the relationship between intramuscular HSP70 abundance and durability is assessed in future studies.

As power output at the moderate-to-heavy-intensity transition declines during prolonged exercise, identification of a marker that can be viewed live during prolonged exercise and used to assess proximity to the moderate-to-heavy-intensity transition would be useful for within-session training intensity regulation (Maunder et al. 2021b). Here we measured muscle oxygenation (S_m_O_2_) using a non-invasive, wireless near-infrared spectroscopy device that could theoretically be used for this purpose. Measures of S_m_O_2_ reflect the balance between local oxygen use and supply (Wittekind et al. 2012; Yogev et al. 2023). The exercise intensity domains show distinct S_m_O_2_ responses to prolonged exercise (Kirby et al. 2021), and the S_m_O_2_ response to incremental exercise can be used to identify intensity domain transitions (Batterson et al. 2023). We found that the S_m_O_2_ coinciding with the moderate-to-heavy-intensity transition was not systematically different between PRE and POST (Fig. 3d). This supports the live monitoring of S_m_O_2_ for within-session intensity regulation, as estimates of the S_m_O_2_ associated with the moderate-to-heavy-intensity transition derived in routine physiological profiling assessments appear to hold over time during prolonged exercise. However, the within-subject CV for S_m_O_2_ at the moderate-to-heavy-intensity transition was ~ 13%, which suggests these measurements should be applied to prolonged exercise with caution. This variability may be due to movement of the device and therefore measurement site (Crum et al. 2017). Nevertheless, our data support further exploration of how S_m_O_2_ can be applied to within-session intensity regulation during prolonged exercise. We recommend that other S_m_O_2_ indices such as deoxygenated haemoglobin are also explored.

This study is limited by the sample size. We may have been insufficiently powered to detect relationships between durability and the intramuscular variables, given the known variability in the assays performed. In a between-subject analysis, this variability is further exacerbated by minor between-subject differences in the biopsy site, given previous research showing variability in intramuscular parameters within an individual at different sites along a muscle tissue (Horwarth et al. 2021). Furthermore, we cannot determine if the results observed within this study readily translate to elite athletes, to prolonged exercise with carbohydrate ingestion, or during stochastic-intensity prolonged exercise protocols, that may be more reflective of real-world road cycling events (Fernández-García et al. 2000; Sanders et al. 2019). We, therefore, recommend that the implications of durability for endurance performance are studied using a range of prolonged exercise protocols, athlete populations, and sports to provide a more detailed understanding of how durability influences real-world endurance performance outcomes.

In conclusion, we observed that durability of the moderate-to-heavy-intensity transition was related to the effect of prolonged exercise on severe-intensity time trial performance. However, we were unable to identify intramuscular variables that related to durability of the moderate-to-heavy-intensity transition. This study provides the first direct support that durability of the moderate-to-heavy-intensity transition is an important endurance performance parameter, and therefore that individual monitoring of durability of the moderate-to-heavy-intensity transition may be valuable.

## Data Availability

Data are available from the corresponding author upon reasonable request.

## Abbreviations

CS: Citrate synthase
CV: Coefficient of variation
EE: Energy expenditure
PFO: Peak fat oxidation rate
S_m_O_2_: Muscle oxygen saturation
r_s_: Spearman’s rank-order correlation coefficient
V̇O_2_: Rate of oxygen consumption
V̇O_2_peak: Peak rate of oxygen consumption
VT_1_: First ventilatory threshold

## Code availability

None used.

## References

[CR1] Areta JL, Hopkins WG (2018) Skeletal muscle glycogen content at rest and during endurance exercise in humans: A meta-analysis. Sports Med 48:2091–2102 29923148 10.1007/s40279-018-0941-1

[CR2] Baguet A, Everaert I, Hespel P et al (2011) A new method for non-invasive estimation of human muscle fiber type composition. PLoS ONE 6:1–6. 10.1371/journal.pone.0021956 10.1371/journal.pone.0021956 PMC3131401 21760934

[CR3] Batterson PM, Kirby BS, Hasselmann G, Feldmann A (2023) Muscle oxygen saturation rates coincide with lactate-based exercise thresholds. Eur J Appl Physiol. 10.1007/s00421-023-05238-9 37261552 10.1007/s00421-023-05238-9

[CR4] Black MI, Jones AM, Blackwell JR et al (2017) Muscle metabolic and neuromuscular determinants of fatigue during cycling in different exercise intensity domains. J Appl Physiol 122:446–459. 10.1152/japplphysiol.00942.2016 28008101 10.1152/japplphysiol.00942.2016 PMC5429469

[CR5] Brownstein CG, Sabater Pastor F, Mira J et al (2022) Power output manipulation from below to above the gas exchange threshold results in exacerbated performance fatigability. Med Sci Sports Exerc. 10.1249/MSS.0000000000002976 36007155 10.1249/MSS.0000000000002976

[CR6] Cairns SP, Renaud JM (2023) The potassium-glycogen interaction on force and excitability in mouse skeletal muscle: implications for fatigue. J Physiol. 10.1113/JP285129support-information-section 37934587 10.1113/JP285129support-information-section

[CR7] Cairns SP, Hing WA, Slack JR et al (1997) Different effects of raised [K+](o) on membrane potential and contraction in mouse fast- and slow-twitch muscle. Am J Phys Cell Physiol 273:C598–C611. 10.1152/ajpcell.1997.273.2.c598 10.1152/ajpcell.1997.273.2.c598 9277357

[CR8] Clark IE, Vanhatalo A, Bailey SJ et al (2018) Effects of two hours of heavy-intensity exercise on the power-duration relationship. Med Sci Sports Exerc 50:1658–1668. 10.1249/MSS.0000000000001601 29521722 10.1249/MSS.0000000000001601

[CR9] Clark IE, Vanhatalo A, Thompson C et al (2019a) Changes in the power-duration relationship following prolonged exercise: estimation using conventional and all-out protocols and relationship with muscle glycogen. Am J Physiol Regul Integr Comp Physiol 317:R59–R67. 10.1152/ajpregu.00031.2019 30995104 10.1152/ajpregu.00031.2019

[CR10] Clark IE, Vanhatalo A, Thompson C et al (2019b) Dynamics of the power-duration relationship during prolonged endurance exercise and influence of carbohydrate ingestion. J Appl Physiol 127:726–736. 10.1152/japplphysiol.00207.2019 31295069 10.1152/japplphysiol.00207.2019

[CR11] Cohen J (1992) A power primer. Psychol Bull 112:155–159 19565683 10.1037/0033-2909.112.1.155

[CR12] Crum EM, O’Connor WJ, Van Loo L et al (2017) Validity and reliability of the Moxy oxygen monitor during incremental cycling exercise. Eur J Sport Sci 17:1037–1043. 10.1080/17461391.2017.1330899 28557670 10.1080/17461391.2017.1330899

[CR13] de Paoli FV, Overgaard K, Pedersen TH, Nielsen OB (2007) Additive protective effects of the addition of lactic acid and adrenaline on excitability and force in isolated rat skeletal muscle depressed by elevated extracellular K+. J Physiol 581:829–839. 10.1113/jphysiol.2007.129049 17347268 10.1113/jphysiol.2007.129049 PMC2075200

[CR14] Febbraio MA, Snow RJ, Stathis CG et al (1994) Effect of heat stress on muscle energy metabolism during exercise. J Appl Physiol 77:2827–2831 7896628 10.1152/jappl.1994.77.6.2827

[CR15] Fernández-García B, Pérez-Landaluce J, Rodríguez-Alonso M, Terrados N (2000) Intensity of exercise during road race pro-cycling competition. Med Sci Sports Exerc 32:1002–1006. 10.1097/00005768-200005000-00019 10795793 10.1097/00005768-200005000-00019

[CR16] Frandsen J, Vest S, Larsen S et al (2017) Maximal fat oxidation is related to performance in an Ironman triathlon. Int J Sports Med 38:975–982. 10.1055/s-0043-117178 29050040 10.1055/s-0043-117178

[CR17] Gonzalez JT, Fuchs CJ, Betts JA, van Loon LJC (2016) Liver glycogen metabolism during and after prolonged endurance-type exercise. Am J of Physiol - Endocrinol and Metab 311:E543–E553. 10.1152/ajpendo.00232.2016 27436612 10.1152/ajpendo.00232.2016

[CR18] Harris RC, Dunnett M, Greenhaff PL (1998) Carnosine and taurine contents in individual fibres of human vastus lateralis muscle. J Sports Sci 16:639–643. 10.1080/026404198366443 10.1080/026404198366443

[CR19] Hopker JG, O’Grady C, Pageaux B (2017) Prolonged constant load cycling exercise is associated with reduced gross efficiency and increased muscle oxygen uptake. Scand J Med Sci Sports 27:408–417. 10.1111/sms.12673 26993076 10.1111/sms.12673

[CR20] Hopkins WG, Schabort EJ, Hawley JA (2001) Reliability of power in physical performance tests. Sports Med 31:211–234 11286357 10.2165/00007256-200131030-00005

[CR21] Horwarth O, Envall H, Röja J et al (2021) Variability in vastus lateralis fiber type distribution, fiber size, and myonuclear content along and between the legs. J Appl Physiol 131:158–173. 10.1152/japplphysiol.00053.2021 34013752 10.1152/japplphysiol.00053.2021

[CR22] Jensen R, Nielsen J, Ørtenblad N (2020) Inhibition of glycogenolysis prolongs action potential repriming period and impairs muscle function in rat skeletal muscle. J Physiol 598:789–803. 10.1113/JP278543 31823376 10.1113/JP278543

[CR23] Jeukendrup AE, Wallis GA (2005) Measurement of substrate oxidation during exercise by means of gas exchange measurements. Int J Sports Med 26:S28–S37. 10.1055/s-2004-830512 15702454 10.1055/s-2004-830512

[CR24] Jones AM, Burnley M, Black MI, et al. (2019) The maximal metabolic steady state redefining the gold standard. Physiol Rep. 10.14814/phy2.14098 10.14814/phy2.14098 PMC6533178 31124324

[CR25] Kirby BS, Clark SA, Bradley EM, Wilkins BW (2021) The balance of muscle oxygen supply and demand reveals critical metabolic rate and predicts time to exhaustion. J Appl Physiol 130:1915–1927. 10.1152/japplphysiol.00058.2021 33914662 10.1152/japplphysiol.00058.2021

[CR26] Krüger K, Reichel T, Zeilinger C (2019) Role of heat shock proteins 70 / 90 in exercise physiology and exercise immunology and their diagnostic potential in sports. J Appl Physiol 126:916–927. 10.1152/japplphysiol.01052.2018 30730806 10.1152/japplphysiol.01052.2018

[CR27] Larsen S, Nielsen J, Hansen CN et al (2012) Biomarkers of mitochondrial content in skeletal muscle of healthy young human subjects. J Physiol 590:3349–3360. 10.1113/jphysiol.2012.230185 22586215 10.1113/jphysiol.2012.230185 PMC3459047

[CR28] Layec G, Blain GM, Rossman MJ et al (2018) Acute high-intensity exercise impairs skeletal muscle respiratory capacity. Med Sci Sports Exerc 50:2409–2417. 10.1249/MSS.0000000000001735 30102675 10.1249/MSS.0000000000001735 PMC6298038

[CR29] Leo P, Spragg J, Podlogar T et al (2022) Power profiling and the power-duration relationship in cycling: a narrative review. Eur J Appl Physiol 122:301–316. 10.1007/s00421-021-04833-y 34708276 10.1007/s00421-021-04833-y PMC8783871

[CR30] Lewis MT, Blain GM, Hart CR et al (2021) Acute high-intensity exercise and skeletal muscle mitochondrial respiratory function: role of metabolic perturbation. Am J of Physiol - Regul, Integr and Comp Physiol 321:R687–R698. 10.1152/ajpregu.00158.2021 34549627 10.1152/ajpregu.00158.2021 PMC8616624

[CR31] Maunder E, Plews DJ, Kilding AE (2018) Contextualising maximal fat oxidation during exercise: determinants and normative values. Front Physiol 9:1–13. 10.3389/fphys.2018.00599 29875697 10.3389/fphys.2018.00599 PMC5974542

[CR32] Maunder E, Plews DJ, Wallis GA, et al (2021a) Temperate performance and metabolic adaptations following endurance training performed under environmental heat stress. Physiol Rep 9:e14849. 10.14814/phy2.14849 10.14814/phy2.14849 PMC8114151 33977674

[CR33] Maunder E, Seiler S, Mildenhall MJ et al (2021b) The importance of ‘durability’ in the physiological profiling of endurance athletes. Sports Med 51:1619–1628. 10.1007/s40279-021-01459-0 33886100 10.1007/s40279-021-01459-0

[CR34] Maunder E, Plews DJ, Wallis GA et al (2022) Peak fat oxidation is positively associated with vastus lateralis CD36 content, fed-state exercise fat oxidation, and endurance performance in trained males. Eur J Appl Physiol 122:93–102. 10.1007/s00421-021-04820-3 34562114 10.1007/s00421-021-04820-3 PMC8475903

[CR35] Maunder E, Rothschild JA, Fritzen AM et al (2023) Skeletal muscle proteins involved in fatty acid transport influence fatty acid oxidation rates observed during exercise. Pflugers Arch 475:1061–1072. 10.1007/s00424-023-02843-7 37464190 10.1007/s00424-023-02843-7 PMC10409849

[CR36] McCormick A, Meijen C, Marcora S (2015) Psychological determinants of whole-body endurance performance. Sports Med 45:997–1015. 10.1007/s40279-015-0319-6 25771784 10.1007/s40279-015-0319-6 PMC4473096

[CR37] Mohr M, Nielsen JJ, Bangsbo J (2011) Caffeine intake improves intense intermittent exercise performance and reduces muscle interstitial potassium accumulation. J Appl Physiol 111:1372–1379. 10.1152/japplphysiol.01028.2010 21836046 10.1152/japplphysiol.01028.2010

[CR38] Morton JP, Kayani AC, McArdle A, Drust B (2009) The exercise-induced stress response of skeletal muscle, with specific emphasis on humans. Sports Med 39:643–662. 10.2165/00007256-200939080-00003 19769414 10.2165/00007256-200939080-00003

[CR39] Nielsen J, Schrøder HD, Rix CG, Ørtenblad N (2009) Distinct effects of subcellular glycogen localization on tetanic relaxation time and endurance in mechanically skinned rat skeletal muscle fibres. J Physiol 587:3679–3690. 10.1113/jphysiol.2009.174862 19470780 10.1113/jphysiol.2009.174862 PMC2742290

[CR40] Nielsen J, Cheng AJ, Ørtenblad N, Westerblad H (2014) Subcellular distribution of glycogen and decreased tetanic Ca2+ in fatigued single intact mouse muscle fibres. J Physiol 592:2003–2012. 10.1113/jphysiol.2014.271528 24591577 10.1113/jphysiol.2014.271528 PMC4230775

[CR41] Nielsen J, Dubillot P, Stausholm MLH, Ørtenblad N (2022) Specific ATPases drive compartmentalized glycogen utilization in rat skeletal muscle. J Gen Physiol 154:1–9. 10.1085/jgp.202113071 10.1085/jgp.202113071 PMC9270182 35796670

[CR42] Ørtenblad N, Nielsen J, Saltin B, Holmberg HC (2011) Role of glycogen availability in sarcoplasmic reticulum Ca2+ kinetics in human skeletal muscle. J Physiol 589:711–725. 10.1113/jphysiol.2010.195982 21135051 10.1113/jphysiol.2010.195982 PMC3055553

[CR43] Ørtenblad N, Westerblad H, Nielsen J (2013) Muscle glycogen stores and fatigue. J Physiol 591:4405–4413. 10.1113/jphysiol.2013.251629 23652590 10.1113/jphysiol.2013.251629 PMC3784189

[CR44] Passfield L, Doust JH (2000) Changes in cycling efficiency and performance after endurance exercise. Med Sci Sports Exerc 32:1935–1941. 10.1097/00005768-200011000-00018 11079525 10.1097/00005768-200011000-00018

[CR45] Paton CD, Hopkins WG (2001) Tests of cycling performance. Sports Med 31:489–496 11428686 10.2165/00007256-200131070-00004

[CR46] Peake JM, Neubauer O, Gatta PAD, Nosaka K (2017) Muscle damage and inflammation during recovery from exercise. J Appl Physiol 122:559–570. 10.1152/japplphysiol.00971.2016 28035017 10.1152/japplphysiol.00971.2016

[CR47] Sahlin K, Shabalina IG, Mattsson CM et al (2010) Ultraendurance exercise increases the production of reactive oxygen species in isolated mitochondria from human skeletal muscle. J Appl Physiol 108:780–787. 10.1152/japplphysiol.00966.2009 20110545 10.1152/japplphysiol.00966.2009 PMC2853199

[CR48] Sanders D, van Erp T, De Koning JJ (2019) Intensity and load characteristics of professional road cycling: Differences between men’s and women’s races. Int J Sports Physiol Perform 14:296–302 30080422 10.1123/ijspp.2018-0190

[CR49] Spragg J, Leo P, Swart J (2023a) The relationship between physiological characteristics and durability in male professional cyclists. Med Sci Sports Exerc 55:133–140. 10.1249/mss.0000000000003024 35977108 10.1249/mss.0000000000003024

[CR50] Spragg J, Leo P, Swart J (2023b) The relationship between training characteristics and durability in professional cyclists across a competitive season. Eur J Sport Sci 23:489–498. 10.1080/17461391.2022.2049886 35239466 10.1080/17461391.2022.2049886

[CR51] Stevens CJ, Mauger AR, Hassmèn P, Taylor L (2018) Endurance performance is influenced by perceptions of pain and emperature: Theory, applications and safety considerations. Sports Med 48:525–537. 10.1007/s40279-017-0852-6 29270865 10.1007/s40279-017-0852-6

[CR52] Stevenson JD, Kilding AE, Plews DJ, Maunder E (2022) Prolonged cycling reduces power output at the moderate-to-heavy intensity transition. Eur J Appl Physiol 122:2673–2682. 10.1007/s00421-022-05036-9 36127418 10.1007/s00421-022-05036-9 PMC9488873

[CR53] Stokie JR, Abbott G, Howlett KF et al (2023) Intramuscular lipid utilization during exercise: a systematic review, meta-analysis, and meta-regression. J Appl Physiol 134:581–592. 10.1152/japplphysiol.00637.2021 36656983 10.1152/japplphysiol.00637.2021

[CR54] Thorstensson A, Karlsson J (1976) Fatiguability and fibre composition of human skeletal muscle. Acta Physiol Scand 98:318–322. 10.1111/j.1748-1716.1976.tb10316.x 136865 10.1111/j.1748-1716.1976.tb10316.x

[CR55] Trewin AJ, Levinger I, Parker L et al (2017) Acute exercise alters skeletal muscle mitochondrial respiration and H2O2 emission in response to hyperinsulinemic-euglycemic clamp in middle-aged obese men. PLoS ONE 12:1–18. 10.1371/journal.pone.0188421 10.1371/journal.pone.0188421 PMC5697830 29161316

[CR56] Vigh-Larsen JF, Ørtenblad N, Nielsen J et al (2022) The role of muscle glycogen content and localization in high-intensity exercise performance: A placebo-controlled trial. Med Sci Sports Exerc 54:2073–2086. 10.1249/MSS.0000000000003002 35868015 10.1249/MSS.0000000000003002

[CR57] Wittekind A, Cooper CE, Elwell CE et al (2012) Warm-up effects on muscle oxygenation, metabolism and sprint cycling performance. Eur J Appl Physiol 112:3129–3139 22212861 10.1007/s00421-011-2262-z

[CR58] Yogev A, Arnold J, Nelson H et al (2023) The effect of severe intensity bouts on muscle oxygen saturation responses in trained cyclists. Frontiers in Sports and Active Living 5:1086227. 10.3389/fspor.2023.1086227 36909360 10.3389/fspor.2023.1086227 PMC9995910

